# Augmented Reality in Medical Education: A Mixed Methods Feasibility Study

**DOI:** 10.7759/cureus.36927

**Published:** 2023-03-30

**Authors:** Oliver George, Jeremy Foster, Zhongyang Xia, Chris Jacobs

**Affiliations:** 1 Medical Education, Great Western Hospitals NHS (National Health Service) Foundation Trust, Swindon, GBR

**Keywords:** teaching technology, augmented reality, simulation, immersion, medical education

## Abstract

Background: Augmented reality (AR) is a novel technology with many applications in medical education. Perhaps one of the most beneficial potential applications is to enable better clinical access for students; however, there is limited research into this use. The purpose of this mixed-methods feasibility study was to evaluate the applicability and acceptability of AR in undergraduate and early postgraduate medical education.

Methods: Single-group quasi-experimental study design was developed for critical care-themed simulation teaching delivered using Microsoft HoloLens (Microsoft Corporation, Redmond, Washington, United States). Post-test questionnaires were completed including a validated adapted immersive experience questionnaire (AIEQ) and an abridged intrinsic motivation inventory (AIMI). The AIMI focused on the domains of ‘interest and enjoyment’, and ‘value and usefulness’. Following the teaching, focus group interviews with thematic analysis were conducted to evaluate participants’ experiences with AR.

Results: All 15 participants (100%) completed the AIEQ and AIMI. Co-located airway teaching (i.e., the demonstrator and participants were placed in the same AR environment) was reported as having a moderate level of user immersion (median 72) and a high level of user enjoyment and value (median 52). Thematic analysis revealed four key themes: visual conceptualization for learning, accessibility, varied immersion, and future application.

Conclusions: Remote simulation for the management of airways in critical care was found to be acceptable and afforded a high level of enjoyment and value. Similarly, this was reflected in the thematic analysis. However, immersion was rated variably in both AIEQ and thematic analysis. The challenges identified with the application of AR included technical infrastructure and patient consent. AR-enabled education benefits are relevant to a number of clinical teaching areas.

## Introduction

Definitions

Augmented reality (AR) is defined as a technology that allows virtual elements to be superimposed onto the user’s perspective of their surroundings. This can be further subdivided into “pass-through AR” where real-world images are captured on camera and displayed by screen to the user (eg. smartphone AR applications/some head-mounted displays (HMDs); and “see-through AR” where the real world is viewed directly through the visor of the HMD with overlaid virtual elements displayed concurrently [1].

Development of AR

Forays into AR date back to the 1960s [2], but initial uptake and application were relatively limited. However, since the 1990s momentum has been building, especially in medical education [1]. Several augmented reality applications (ARAs) are now commercially available, one of which is the Microsoft HoloLens (currently in its second generation) (Microsoft Corporation, Redmond, Washington, United States).

A pilot study in anatomy training using HoloLens demonstrated significant potential for medical education [3], and Case Western Reserve University's development in this area has shown a positive learning experience and a time-efficient modality, which presents potential cost savings to institutions [4].

Three integrative reviews have suggested that there has been a lack of learning theory behind its use [5-7], despite potential underlying learning factors that have previously been identified, for example, thinking of AR as a scaffolding tool in a social constructivist model of learning [8]. In an initial literature review, only one article was found to explicitly outline a theory-based framework for use of AR in education (specifically a mobile AR app) [7]. In a 2022 scoping review by Jacobs et al., a narrative synthesis of head-mounted AR in medical education showed benefit [5]; however, studies evaluated using the Medical Education Research Study Quality Instrument (MERSQI) identified a common pattern of studies not describing a validated instrument to measure participant performance or experience [7].

AR in medical education

AR has an increasing role in medical education with various functions, including creating an immersive environment and authentic participatory reality [9], and intuitive uses, including teaching anatomy [6,10]. However, there is conflicting evidence as to the effectiveness of AR for theoretical learning compared to traditional textbook methods in studies using mobile AR apps [11,12]. It is also unclear whether any perceived benefits are due to novelty rather than any true increase in teaching effectiveness [11,13]. Moro et al. found no significant differences in brain physiology and anatomy knowledge acquisition between HoloLens and a mobile AR application [9].

AR also fosters a safe environment to make and learn from errors [14]. This is extremely helpful for undergraduate and postgraduate surgical simulation, where the utility [15] and acceptability [16] of AR have been highlighted; research on AR in surgical training makes up most of the AR for medical education literature [17].

An extensively evaluated area of AR application is teaching procedural skills, at both the undergraduate and postgraduate levels. AR was found to standardise performance and ensure fewer procedural errors in a study comparing conventional and AR methods of teaching extracorporeal membrane oxygenation (ECMO) cannulation [12]. Gerup et al.’s integrative review of AR for healthcare education showed several trials of AR to teach central venous cannulation (CVC) [6]. For example, Rochlen et al.’s 2017 pilot study demonstrated the usability and feasibility of see-through AR for this procedure and was specifically useful in visualising the anatomy [18].

Learning Opportunity

Potentially one of the most beneficial applications for AR in medical education is to enable better clinical access for students. Using the technology for “a remote access mixed reality teaching ward round”, Bala et al. showed in their proof-of-concept study how AR may be used to deliver more equitable, standardised, insightful and enjoyable clinical teaching for those traditionally hard-to-access areas [19]. There is limited research into this AR use and this study aims to add to this field.

Current medical students have been significantly affected by the lack of clinical exposure during the coronavirus disease 2019 (COVID-19) pandemic, which has exacerbated the perceived inequality of learning opportunities between, or even within, cohorts. Students themselves are often keen to drive forward innovative learning methods, even more so with the disruption of COVID-19 [20].

When fully utilised, clinical placement promotes situated learning, where students engage with the team, its culture, norms, and practices in a two-way relationship. They progress to becoming legitimate peripheral participants in that area [21]. However, this process can be impaired due to a lack of capacity for students in certain hard-to-access areas, or due to restrictions such as those seen with the COVID-19 pandemic.

AR could be used to provide an alternative to in-person clinical placement, through an interactive, point-of-view, virtual graphic-enhanced, live feed of simulated or real clinical scenarios. In this way it would build on its accepted social constructivist merits as a more knowledgeable educator enables learners to build on their prior theoretical knowledge, applying it to practice: clinical scenarios [8,22].

Taking an experiential view of learning, AR could be instrumental in the “re-learning” of ideas, previously learnt in the classroom, but now experienced in (augmented) reality. This mode of learning could better reflect the real world in which ideas are applied, emphasising the crucial two-way relationship between the person and their environment [23]. With the development of these ideas into 21st-century “experience-based learning”, AR could be promising in nurturing “supported participation” (the key factor in effective workplace learning), especially when easing learners into clinical placement at a safe, but high-fidelity, distance [24]. 

Research aim

This feasibility study has been designed to evaluate the potential utility and acceptability of AR in undergraduate and early postgraduate medical education for clinical areas that are harder to gain exposure to. Our study uses simulation to demonstrate this. Furthermore, using validated measures we aim to capture the user experience of AR as a tool for evaluation.

## Materials and methods

Participants

Convenience sampling occurred at the research institution and 15 participants were recruited: this included 13 senior clinical medical students at United Kingdom (UK) universities and two newly qualified doctors. Participants’ age range was 22 to 36, with 10 females and five males. All participants had previous simulation experience. Written consent was obtained at enrolment, and this was stored securely in line with the General Data Protection Regulations United Kingdom (GDPR).

Eligibility and exclusion criteria

Criteria were set to promote the inclusion of those wishing to gain education in critical care. However, to preserve study group homogeneity, an exclusion criterion of $\geq$4 months for critical care placement was set. No participants met this requirement, and thus all 15 were included. Table 1 completes the full eligibility criteria.

**Table 1:** Study eligibility criteria.

Inclusion criteria	Exclusion criteria
Any senior clinical medical student (either fourth or fifth year) on placement at the hospital trust	Critical care placement ≥4 months
Any foundation year doctor currently working at the trust	

Setting

This was a single site (Great Western Hospital, Swindon) and a single group study. The teaching session used the high-fidelity, purpose-built simulation suite and a neighbouring seminar room in the education centre.

Study process

**Figure 1:**
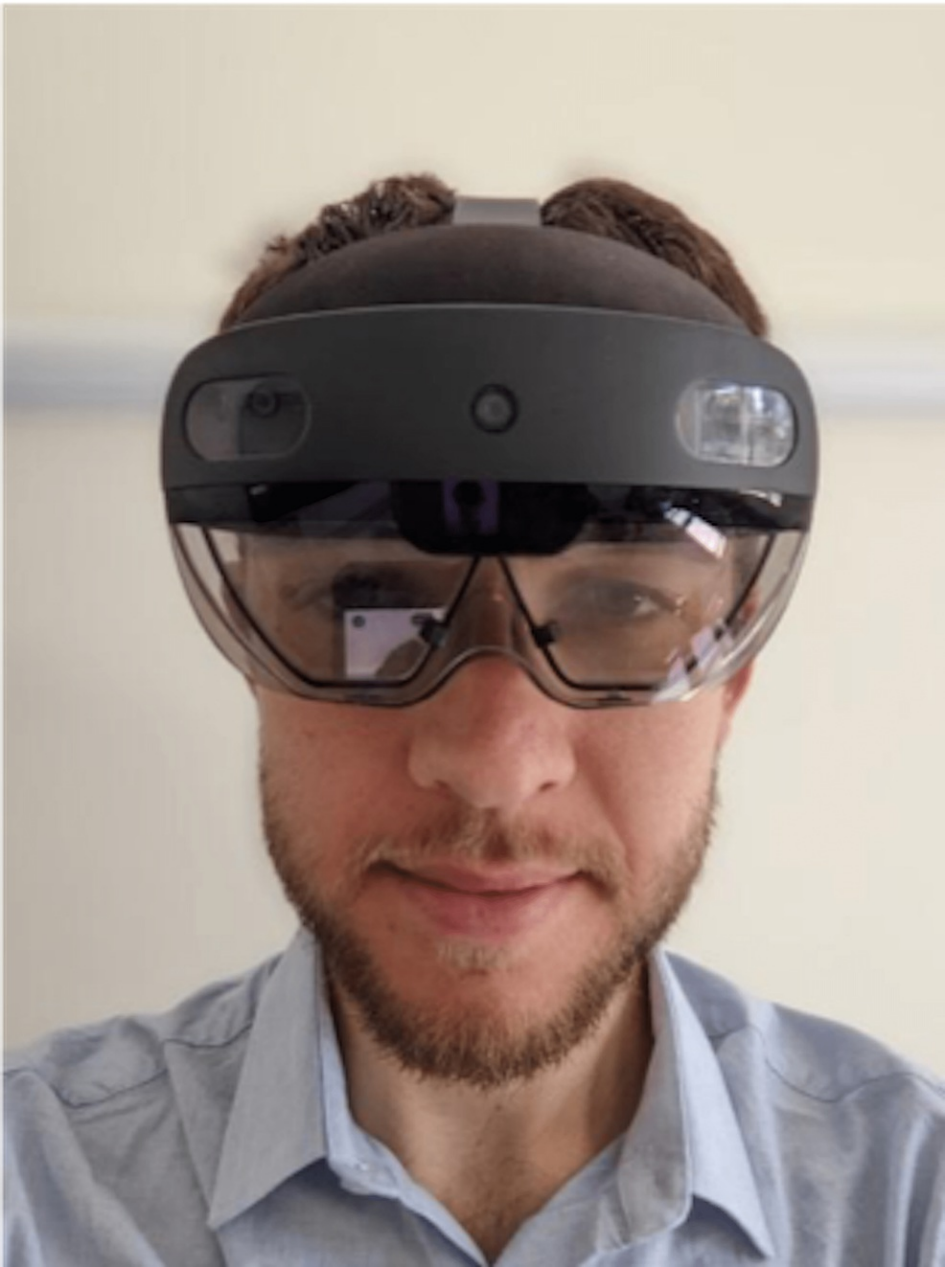
The Microsoft HoloLens* on the demonstrator. *Microsoft Corporation, Redmond, Washington, United States The demonstrator in the photograph is one of the authors

**Figure 2:**
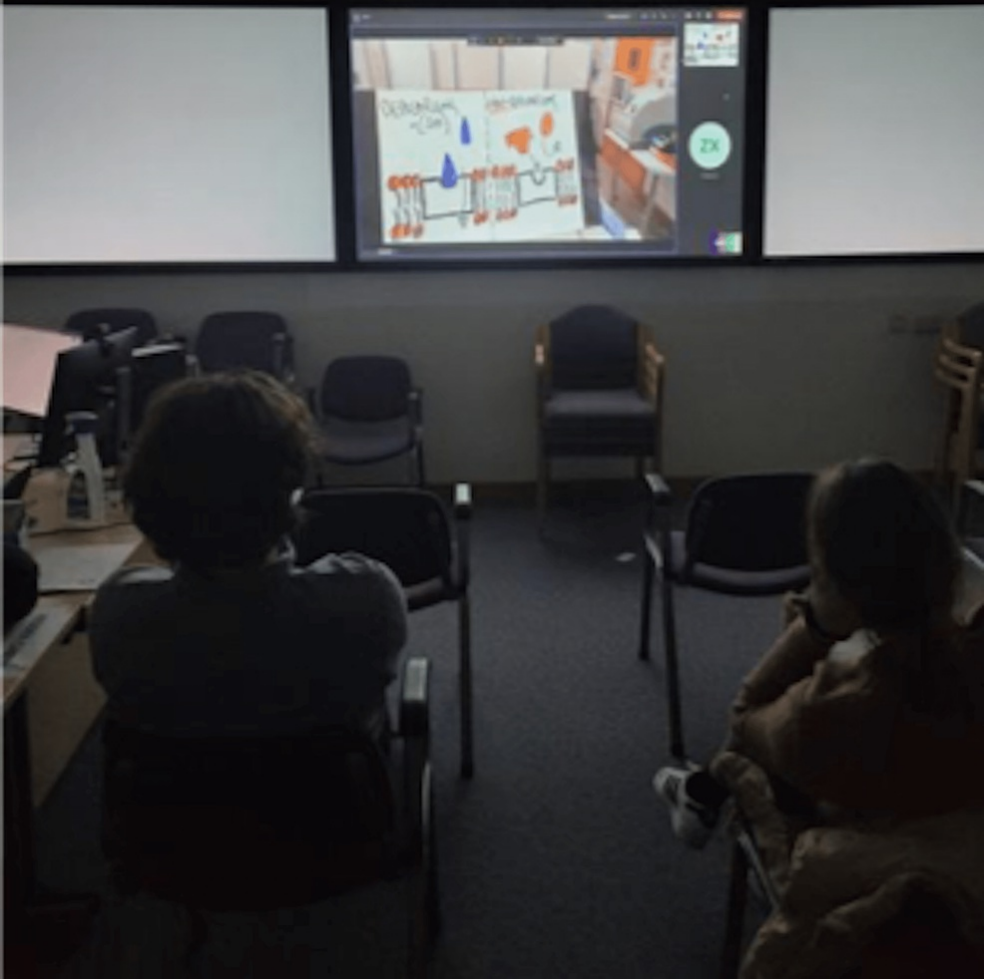
Participants in the neighbouring seminar room.

**Figure 3:**
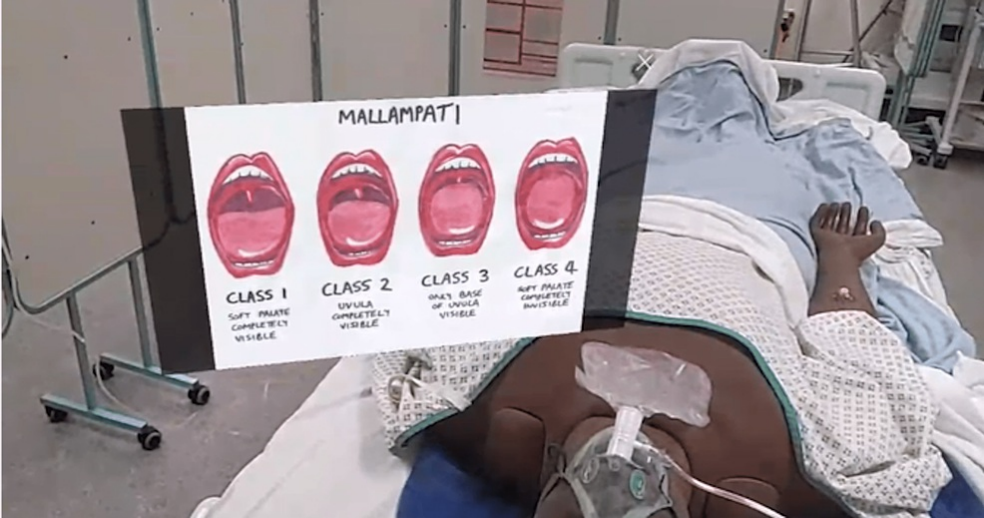
Augmented reality overlay being used to help clarify teaching points. Here, it is the Mallampati score.

**Figure 4:**
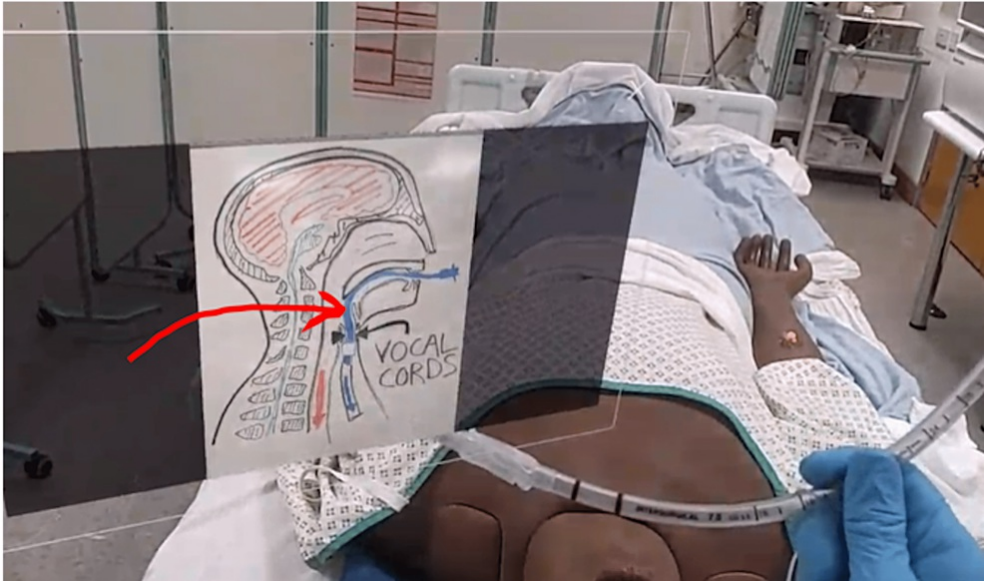
Augmented reality overlay with an arrow being used to help clarify a teaching point. This used the Microsoft Teams Remote Assist* interface. *Microsoft Corporation, Redmond, Washington, United States

The 15 participants were divided into two groups, with two smaller group teaching sessions delivered on separate dates by the same facilitators. Both groups received the same 20-minute critical care-themed teaching session delivered using Microsoft HoloLens (2nd generation), an AR HMD, with Microsoft Remote Assist, Microsoft Teams, and Microsoft PowerPoint. The session content was created in-house by the authors, and reviewed by a senior anaesthetist before implementation. In the session, the demonstrator wore the Microsoft HoloLens (Figure 1) and delivered a teaching session on rapid sequence intubation from the simulation suite. The participants watched a livestream in the neighbouring seminar room via Microsoft Teams (Figure 2). They were able to see the same point-of-view visuals as the demonstrator and could interact with the demonstrator in turn, asking questions in real time. AR was used to help clarify difficult physiology, pharmacology, and anatomy through related images (Figures 3, 4). These images could be moved and enlarged by the demonstrator, and they could also draw on the images using tools in the Microsoft Teams Remote Assist interface (Figure 4). 

A technician was present in the simulation suite to help the demonstrator with technical aspects of the teaching session, such as finding and positioning equipment.

Outcome measures

Quantitative Data and Statistical Analysis Plan

Quantitative data was collected through an adapted immersive experience questionnaire (AIEQ) and an abridged intrinsic motivation inventory (AIMI). The AIMI focused on the domains of interest and enjoyment, and value and usefulness. Both the AIEQ and AIMI were adapted from research by Jacobs and Rigby, who have explored validation in these immersion and motivation surveys for healthcare education [25].

Items on each questionnaire were Likert-scaled, and the maximal Likert score for each item was five. Likert-scaled data was assumed to be continuous. The AEIQ included 21 items, the AIMI domain of interest and enjoyment included seven items, and the AIMI domain of value and usefulness had five items. Following a validation study of the AIMI for the clinical teaching environment [25], this study reduced the Likert scale from seven to five to match the AIEQ. In addition, the AEIQ included a self-reported immersion (SRI) score rated out of 10. Descriptive statistical analysis was undertaken to determine medians and spread of data, and the strength of linear association between outcomes was calculated using Pearson correlation with a high degree of variable correlation if the value is >0.50. For statistical analysis, Microsoft Excel (version 16) and online software (socstatistics.com) were used.

Qualitative Data

In addition, five small group semi-structured interviews were conducted for hybrid thematic analysis. Groups were sized at three to four participants and within each group, all participants were in a similar year of study or grade. Interviews were conducted by two authors (OG and JF), transcribed verbatim (by either OG, JF, or ZX), and independently coded by each author (OG, JF, and ZX). An open coding method was used and codes were collated into themes and reviewed by the three co-authors together (OG, JF, and ZX). Following this, the final thematic schema was developed (OG, JF, and ZX), and this was reviewed by fellow author CJ. This thematic analysis process was based on Braun and Clarke’s six-step approach [26].

Ethical approval

Ethical approval was given by the Swindon Academy Medical Education Research Committee in November 2021 (approval number: OG051121).

## Results

AIEQ and AIMI

**Figure 5:**
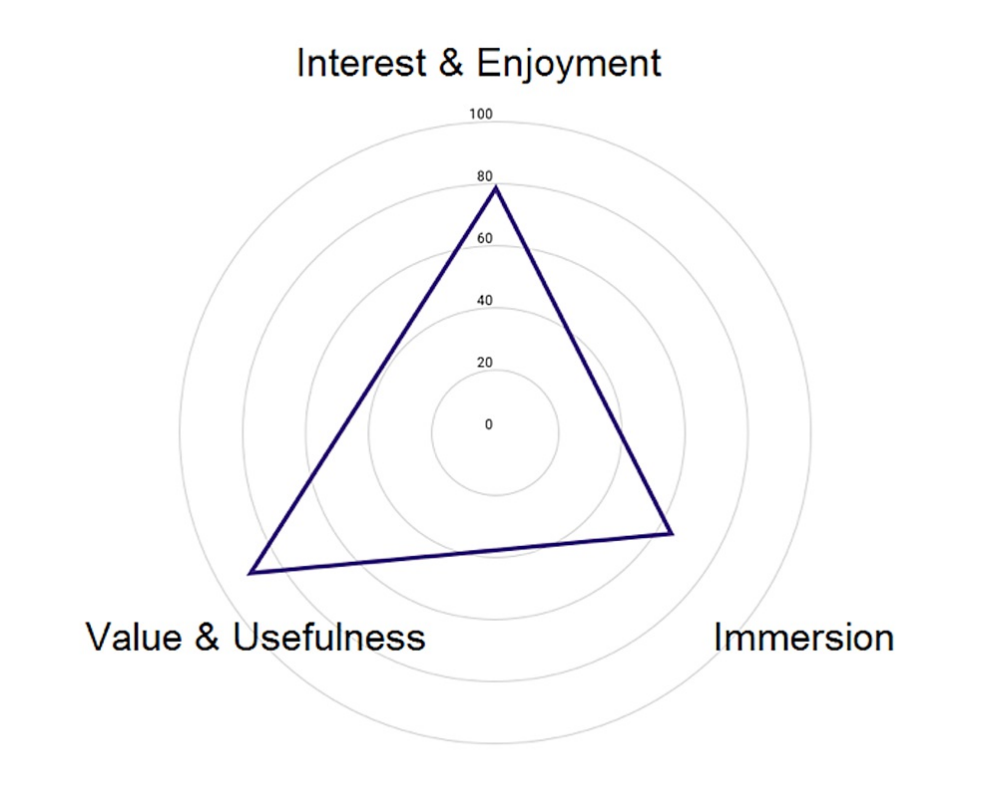
Comparison between the "Interest and Enjoyment" and "Value and Usefulness" domains from the AIMI, and the total Immersion domain from the IEQ. Mean scores have been converted to percentage (n=15). AIMI: abridged intrinsic motivation inventory; IEQ: immersive experience questionnaire

A test for normality demonstrated non-normally distributed data and scores were reported as medians. The AIEQ was scored as previously described, and the total immersion (TI) score was the sum of the AIEQ items. The AIEQ median score was 72.00 (IQR 16.5). The median SRI score was 6.00 (IQR 3). A high correlation existed between the TI score and the SRI (rs = 0.76, n = 15, p <0.001). The median score for the AIMI domain of interest and enjoyment was 28.00 (IQR 7.5). The median score for the AIMI domain of value and usefulness was 24.00 (IQR 4). Descriptive findings of both the AIEQ and AIMI findings have been illustrated in Table 2. A visual representation of averaged AIEQ and AIMI scores has been made in Figure 5. 

**Table 2:** Descriptive findings from AIEQ and AIMI. SRI: self-reported immersion; AIEQ: adapted immersive experience questionnaire; AIMI: abridged intrinsic motivation inventory

	Total immersion score	SRI	AIEQ (total immersion score + SRI)	AIMI interest and enjoyment	AIMI value and usefulness
Total dataset (n)	15				
Maximum score	100	10	110	35	25
Standard deviation	9.71	2.33	11.58	5.23	3.46
Upper quartile	71	7	77.50	31.50	25
Median	66	6	72	28	24
Lower quartile	57	4	61	24	21
Interquartile range	14	3	16.5	7.5	4

Thematic analysis

The main thematic analysis findings have been highlighted below, broken down into key themes. Within these themes, details and quotes have been provided to give context. Four key themes were identified.

Theme 1: Ability to Visualise Difficult Concepts

Interviewees felt the HoloLens was able to improve the ability to visualise difficult concepts. It is able to overlay annotated diagrams into the same visual field as the simulation scenario, and this can help improve learner understanding.

"*…really helpful to have the theoretical and the practical applications happening simultaneously…"​*

They are able to see physiology, pharmacology and anatomy embedded into a live clinical situation, and can clarify understanding through two-way questioning. Interviewees noted that having theory and practice run simultaneously, rather than separately, was beneficial to their learning. Equally, they felt it made it more memorable.

"It puts what you would have learnt on paper…suxamethonium is this and it acts in this way…but actually seeing it being used is much more memorable."

Often, medical education is split into two dichotomous stages: preclinical and clinical education. Preclinically, learners are repeatedly overwhelmed with seemingly abstract concepts, and this can seem far removed from clinical medicine. Interviewees identified the Microsoft HoloLens as a useful bridging adjunct, helping to convert preclinical concepts into practice. They stated that it would be useful before going onto the wards.

"…converting all the theory of what we had learnt before into something practical...that would have been super useful before going onto the wards..."

Theme 2: Immersion

Interviewees also commented on immersion, which was rated variably. This was similar to the AIEQ. Some found it very immersive and said it was perhaps as close to in-person teaching as you could possibly get because it was able to transfer high levels of fidelity and realism.

"…seeing someone in front of you, that’s immersive…and that is as close to real life as we could realistically get, I think."

"I didn’t find it that immersive because…you haven’t got your own headset…"

Theme 3: Accessibility

A further theme that emerged from the thematic analysis was accessibility. The HoloLens would improve access to teaching because learners could log in to Microsoft Teams remotely and partake in the session. It would still retain its interactivity, provided learners had access to a microphone. Many interviewees linked this potential to improving accessibility to the COVID-19 pandemic: they could learn at home and wouldn’t have to compromise on session quality.

"Everyone in the room is experiencing the same thing. In big group teaching often there is a crowd of people and you can’t actually see what you are meant to be seeing."

In addition, interviewees noted the teaching session could be scaled up easily, and a large number of learners could attend. Although they felt this may not be as personal, they did feel it would help with teaching standardisation.

"It is quite good in democratising the experience, that different medical students have."

Moreover, the thematic analysis revealed that many interviewees recognised the application of the HoloLens in limited exposure clinical areas. For instance, in large group teaching, learners may be crowded around a bedspace trying to see a procedure, yet because of the number of learners they are unable to see anything. In fact, in any limited space area, they thought the HoloLens would be beneficial, and they gave examples including intensive care, surgery and obstetrics.

“(on future applications) clinically, I just think of any…clinical area where there is limited space or limited access."

The reason behind this benefit was almost entirely attributed to the point-of-view camera angles, which provide the same visuals for both the demonstrator and learner. In some circumstances, namely surgery, interviewees believed it could even replace in-person teaching, which can be too difficult to access. However, one interviewee did express concern about the idea that in-person teaching may be reduced due to improved teaching technology.

Theme 4: Future

The final theme encompassed future challenges with the HoloLens. Interviewees felt that if it was used in the ward on real patients, the headset would be jarring and odd for them. Patient perception was not the only barrier to ward transition, and consent and confidentiality issues were also identified.

"It’d probably be quite jarring for the patient to have their doctor be wearing that headset and videoing hand movements that they can’t see what’s going on."

In addition, there are further considerations that interviewees thought should be recognised. These included training clinicians and/or learners to wear the headset, refinements in the AR functionality and calibration of the hardware, and system improvements such as improved internet connectivity.

## Discussion

The aim of this study was to explore the application of AR in undergraduate (and early postgraduate) medical education for limited exposure clinical areas.

High-scoring Likert items in the AIMI indicate participants believe that AR has interest, enjoyment, value and usefulness as an educational tool. Positive personal experiences with AR have already been documented in medical education. Aebersold et al. found participants enjoyed AR and thought its application was useful when trying to place a nasogastric tube [27], and Bork et al. noted participants found AR fun in integrated radiology teaching [28]. A 2021 systematic review by Parsons and MacCallum further supported this, and they commented that participant experience with AR is generally very positive [29]. Additionally, this review identified a role for AR to enhance learning with limitations in trainees viewing anatomy with traditional simulation mannequins. The findings in this study support these affordances of AR in complex training scenarios.

The intrinsic motivation inventory domains scored highly; previous technology-enhanced “participatory simulations” were shown to be highly intrinsically motivating with two different types of technology (basic and more advanced) [30]. Whether this is entirely due to the simulation and technology itself or the cooperative aspect remains to be seen. Intrinsic motivation was investigated in a randomised control trial comparing 360-degree media viewed in an HMD or on a traditional monitor, which suggested significantly higher motivational scores in viewing the same video in 360-degree format [31]. High reported intrinsic motivation is promising as it has long been linked to high-quality learning, especially of complex concepts such as those found in medicine [32].

However, immersion (defined by the connection to the AR world), was a much more variable entity: in both the AIEQ and thematic analysis there were conflicting opinions. In educational environments, it has been proposed that immersion can enhance learning through perspective, situated experience and skill transfer [33]. Yet, research into immersion and empirical evidence to support this proposition remains in infancy, and a study to evaluate immersion and students’ conceptual understanding found that although low-level immersion does improve participants’ conceptual understanding, a deeper, total immersion has a much weaker association [34]. This study of science students did demonstrate an association between domain-specific motivation and cognitive motivation. Alternative studies primarily addressing immersion in video gaming agreed: a deeper, more total immersion has implications for engagement [35]. In the randomised trial where participants were allocated to either a 360 or a two-dimensional (2D) screen and watched a clinical consultation, AIEQ scores were significantly lower in the 2D format [31]. Additionally, in early work on immersive experience questionnaire (IEQ) by Rigby et al., there was an association between reported immersion to media and screen size [36].

It is still useful, however, to try and understand the reasons behind the variable immersion experienced by participants’ in this study. A reality-virtuality continuum helps delineate between AR and VR, with MR as an overarching definition [37]. In a conceptual revisit to this original continuum, the goal was to integrate display and the potential for incongruence of content [38]. For instance, although some senses such as sight may be immersive, others such as sound, touch and haptic reality may not. This incongruence can be applied to this study: participants may have been immersed in sight and sound, but they are unlikely to have been immersed in touch or haptic reality. Some participants did not even feel immersed in sound, which they attributed to a lack of background noise. High-quality environmental fidelity can positively improve immersion, and AR in the clinical environment would clearly improve this; however, delivering the teaching session in the simulation suite allowed for AR development, refinement and a more flexible delivery [39].

In addition, all our participants watched the augmented session from the same room, and at times our graphics became pixelated because of connectivity. Not only may this have negatively affected immersion, but also the phenomenon of place illusion, which can be both limited and linked by immersion. Place illusion is the sense of being in a place you know you are not, and uses a cave analogy to explain this: two gamers may both be immersed in a virtual reality cave, but if one goes near the walls of the cave and the visuals become pixelated, place illusion may break [40]. Both immersion and place illusion highlight the limitations of current AR technology: HoloLens or AR product development stage and the necessary information technology planning and infrastructure.

The benefit of AR in improving access to learning opportunities seemed important to participants. A study of anatomy learning recognised the ability of AR teaching to be used remotely [41]. A collaborative AR anatomy teaching environment was created for co-located learners, who found it both useful and enabling. The authors proposed that co-located learning through AR may even be preferential to singular learning because it enables a deeper level of understanding.

Limitations

This is a feasibility study and thus, by definition, it is small-scale and designed to give preliminary results, which requires caution in interpreting the findings. The study was a single group without a comparison, leaving the AIEQ and AIMI vulnerable to bias. Additionally, the AIMI Likert scale was amended to 5 points from 7, which impacts the validity and reliability of comparison with other AIMI-based studies. However, limiting the response anchors to five can improve measuring the experience by participants and this aligns both measures to the response scale [42]. 

Moreover, only one topic was covered in the teaching session. Although it did represent a limited exposure clinical scenario and allowed for teaching on anatomy, physiology, and pharmacology with AR supplementation, if participants had an already established interest (or disinterest) in the topic it could have influenced the outcomes and acceptability. Future work to compare different educational settings, content and reality-virtuality type would benefit the medical education community in appraising the utility of immersive technology; for instance, a follow-up study comparing an AR headset to a virtual reality (VR) headset (such as Meta Quest, Meta Platforms, Inc., Menlo Park, California, United States). Much of the data from other studies on AR in medical education similarly follows a feasibility or pilot study design. Currently, there is very little comparison of AR (including HMDs) to conventional methods, particularly in teaching locations that are hard to access.

Participants were not able to wear the Microsoft HoloLens in this study because we wanted the demonstrator to share and explain their point-of-view; this allowed the expert to guide them through the simulation. Moreover, considerable training and experience are needed to operate the Microsoft HoloLens, and we only had one headset. Additional headsets would incur a significant cost. 

## Conclusions

In this feasibility study, we have shown that using AR to teach medical concepts in limited exposure clinical areas is perceived as valuable and useful by learners, with particular strengths in improving equality of access to the clinical learning environment and bridging the theory to clinical practice gap.

Participants found this mode of teaching interesting and enjoyable but rated the immersive experience variably. They outlined several challenges for the future development of AR in medical education such as the technical infrastructure needed and issues around patient consent and perception of AR devices. There is a growing framework linking AR with learning theory in the literature, and this work contributes to this and provides future avenues for research.
